# Sphenoid sinus microbiota in pituitary apoplexy: a preliminary study

**DOI:** 10.1007/s11102-017-0823-9

**Published:** 2017-08-29

**Authors:** Gavin J. Humphreys, Mueez Waqar, Andrew J. McBain, Kanna K. Gnanalingham

**Affiliations:** 1 School of Health Sciences, Faculty of Biology, Medicine and Health, The University of Manchester, Manchester, UK; 2 Manchester Academic Health Sciences Centre (MAHSC), The University of Manchester, Manchester, UK; 3 Department of Neurosurgery, Manchester Centre for Clinical Neurosciences, Salford Royal NHS Foundation Trust, Stott Lane, Salford, M6 8HD UK

**Keywords:** Pituitary apoplexy, Sphenoid sinus, Pituitary adenoma, Microbiota

## Abstract

**Purpose:**

There is a high incidence of abnormal sphenoid sinus changes in patients with pituitary apoplexy (PA). Their pathophysiology is currently unexplored and may reflect an inflammatory or infective process. In this preliminary study, we characterised the microbiota of sphenoid sinus mucosa in patients with PA and compared findings to a control group of surgically treated non-functioning pituitary adenomas (NFPAs).

**Methods:**

In this prospective observational study of patients undergoing trans-sphenoidal surgery for PA or NFPA, sphenoid sinus mucosal specimens were microbiologically profiled through PCR-cloning of the 16S rRNA gene.

**Results:**

Ten patients (five with PA and five with NFPAs) with a mean age of 51 years (range 23–71) were included. Differences in the sphenoid sinus microbiota of the PA and NFPA groups were observed. Four PA patients harboured *Enterobacteriaceae* (*Enterobacter* spp., N = 3; *Escherichia coli*, N = 1). In contrast, patients with NFPAs had a sinus microbiota more representative of health, including *Staphylococcus epidermidis* (*N* = 2) or *Corynebacterium* spp. (*N* = 2).

**Conclusions:**

PA may be associated with an abnormal sphenoid sinus microbiota that is similar to that seen in patients with sphenoid sinusitis.

## Introduction

Pituitary apoplexy (PA) is an acute clinical syndrome secondary to haemorrhage and/or infarction of a pituitary adenoma [1]. Patients classically present with a sudden onset of headache, nausea/vomiting, visual disturbance and/or altered conscious level [1]. PA is presumed to be a spontaneous vascular event and risk factors include anticoagulant therapy, major surgery, dopamine agonist therapy, pregnancy and radiation therapy [1].

We and others have observed a high incidence of abnormal sphenoid sinus changes in PA [2]. In a recent study we noted that sphenoid sinus mucosal thickening (SSMT) was present in 62% of PA patients compared to just 6% of a control group of non-functioning pituitary adenomas (NFPAs) [2]. The aetiology of SSMT in PA is unknown and possibilities include inflammation or infection.

In this preliminary study, we sought to characterise the sphenoid sinus mucosal microbiota in patients with PA and compare them to a control group comprising surgically treated NFPAs.

## Methods

This was a prospective, single-center observational study. Ethical approval was obtained from the Local Research Ethics Committee for the collection of sinus biopsies between 2008 and 2009 (08/H1012/50). Patient data on clinical presentation, preoperative magnetic resonance imaging findings, tumour histology, treatment and follow-up were collected from a prospective electronic database.

### Patient cohorts

Patient cohorts were determined according to the following criteria:

(i) Classical PA: as defined by the Society of Endocrinology, refers to a *clinical syndrome, characterised by a sudden onset of headache, vomiting, visual impairment and decreased consciousness caused by haemorrhage and*/*or infarction of the pituitary gland* [1]. Haemorrhage and/or infarction were confirmed using at least one or more of the following: imaging (T1/2 weighted magnetic resonance imaging), histopathology or intra-operative findings (i.e. evidence of pre-existing haemorrhage within the pituitary adenoma).
(ii) Subclinical PA: defined as radiological and histopathological evidence of infarction or haemorrhage, without accompanying symptomatology.
(iii) NFPA: surgically treated control group, with no evidence of pituitary apoplexy clinically, radiologically or on histopathology.

### Tissue collection

Sphenoid sinus mucosal samples were collected during the approach for endoscopic trans-sphenoidal surgery for the treatment of PA or NFPAs, as described previously [3]. On induction of general anesthesia all patients received a perioperative dose of antibiotics (1.5 g cefuroxime and 500 mg metronidazole; iv). Following surgery, tissue samples were archived at −80 °C for further microbiological analyses.

### Microbiological profiling

Bacterial genomic DNA was extracted from tissue biopsy samples (<25 mg) using a Qiagen DNA Blood and Tissue Kit (Qiagen Ltd, UK), according to the manufacturing protocol. Polymerase chain reaction of extracted bacterial DNA was performed using the 806R/8FLP primer set [4] and amplified products purified using a Qiagen PCR purification kit (Qiagen Ltd, UK). Amplicons were ligated into a pDrive cloning vector (Qiagen Ltd, UK) and transformed into competent Escherichia coli as per the manufacturer’s protocol. Negative controls comprised PCR grade water alone in the absence of amplicon product. Transformants were plated onto Luria–Bertani media (supplemented 0.1 mg/ml ampicillin; 0.05 mM IPTG; 0.08 mg/ml X-gal) and successful transformants identified using blue/white screening. Plasmid DNA purification was performed using the QIAprep Miniprep kit (Qiagen Ltd, UK) and inserts screened by PCR using the vector specific primers M13 forward (5′ GTTTTCCCAGTCACGAC 3′) and M13 reverse (5′ AACAGCTATGACCATG 3′). Inserts were sequenced using M13-vector primers at the University of Manchester Sanger sequencing facility. Consensus sequences were identified following a BLAST search of the GenBank nucleotide database (http://blast.ncbi.nlm.nih.gov/Blast.cgi). Isolates exhibiting >98% homology to database sequences were delineated to the species level.

## Results

**Fig. 1:**
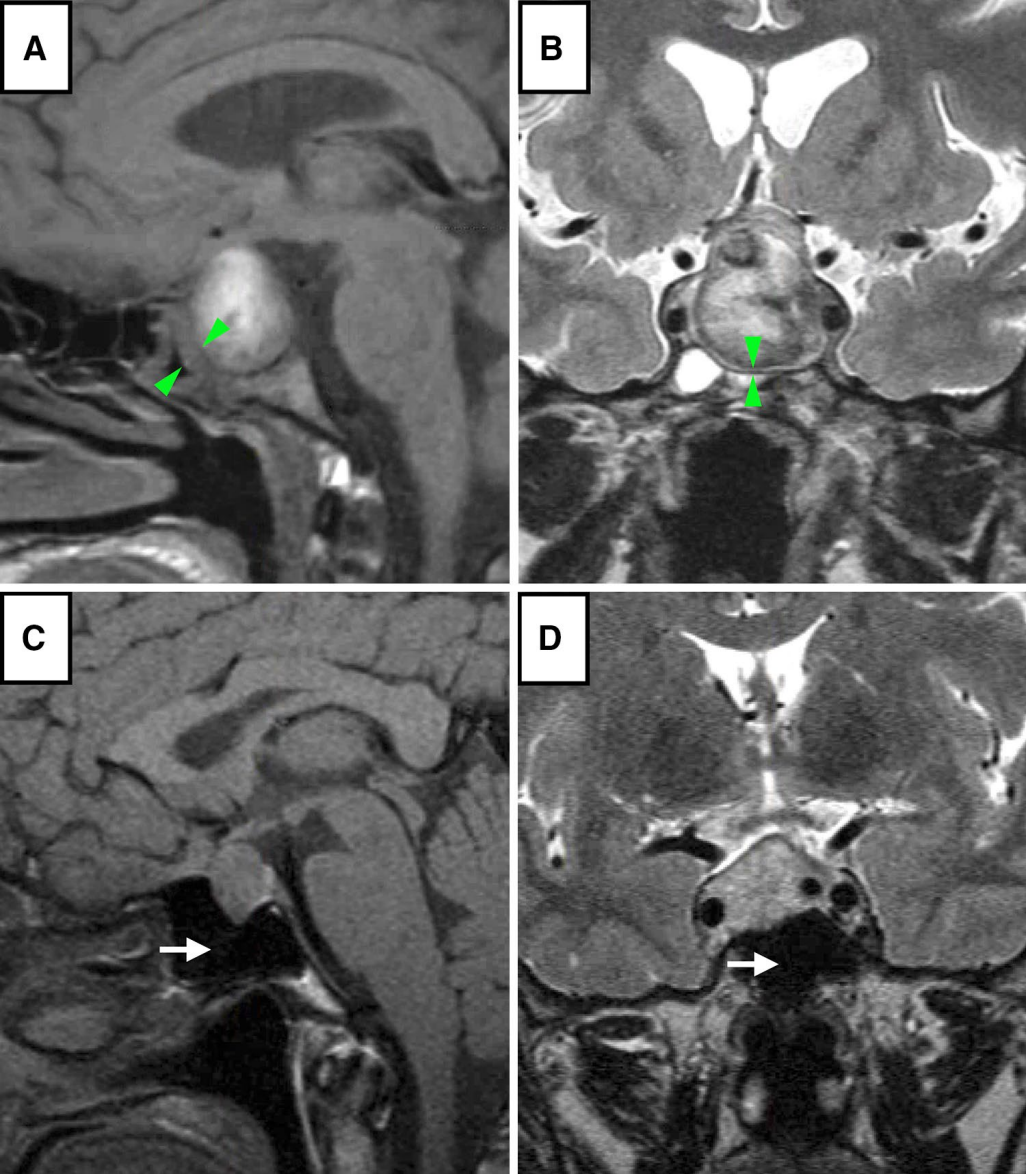
Sagittal T1 (**a** and **c**) and coronal T2-weighted (**b** and **d**) MRI from a patient presenting with PA (**a** and **b**) and NFPA (**c** and **d**). The patient with PA (**a** and **b**; Case A1, Table 1) was a 24 year old male, with no significant past medical history, who presented with acute confusion, headaches and hyponatraemia. MRI revealed a pituitary lesion with evidence of bleed and sphenoid sinus mucosal thickening (*green arrows*). The patient with NFPA (**c** and **d**; Case N3, Table 1) was a 54 year old female who presented with a homonymous hemianopia. MRI revealed a pituitary lesion abutting the optic chiasm and with no evidence of sphenoid sinus mucosal thickening and a relatively empty sinus (*white arrows*)

Ten patients were included in this pilot study, five with PA (four classical and one subclinical) and five with NFPAs (Table 1). The mean age was 51 years (range 23–71). There were four males and six females. The typical MRI scan of a patient with PA (Case A1) and NFPA (Case N3) are shown in Fig. 1.

**Table 1:** Sphenoid sinus microbiota in patients with PA (A1–A5) and NFPAs (N1–N5)

Patient	Demographic	Clinical features	Imaging	Histology*	Management		Microbiology	
					Surgery/oncology	Hormone replacement	Gram positive	Gram negative
A1	24, M	Confusion (GCS 14), low sodium	Mixed signal on T1 MRI with SSMT	Null cell with necrosis	TSS	Steroids, thyroxine	*Lactococcus* sp. *Leuconostoc citreum*	*Enterobacter* sp. *Enterobacter aerogenes*
A2	64, F	Asymptomatic	Cystic high signal on T1 and T2 MRI	LH/FSH with haemorrhage	TSS + XRT	Steroids, thyroxine	*Leuconostoc citreum* *Leuconostoc fallax*	*Stenotrophomonas maltophilia*
A3	58, M	Headache, bitemporal hemianopia	Cystic mixed (high/low) signal on T1 and T2	LH/FSH	TSS	Steroids	*Lactococcus* sp. *Leuconostoc citreum*	*Acinetobacter* sp. *Enterobacter* sp.
A4	23, F	Headache, irregular menses	Cystic high signal lesion on T1; low signal on T2	TSH with haemorrhage	TSS	Nil	*Bacillus* sp. *Corynebacterium fastidiosum* *Lactobacillus casei* *Lactobacillus rhamnosus* *Staphylococcus epidermidis*	*Enterobacter* sp.
A5	61, F	Headache, reduced left sided visual acuity	High signal on T1 and T2 with SSMT	LH/FSH with haemorrhage	TSS + XRT	Steroids, thyroxine, sex hormones, DDAVP	*Corynebacterium propinquum* *Corynebacterium pseudodiphthereticum* *Staphylococcus* sp.	*Escherichia coli*
N1	65, M	Hypo-pituitarism	Iso-signal on T1 and T2	LH/FSH	TSS	Steroids, thyroxine, growth hormone	*Streptococcus* sp. *Lactobacillus delbruecki*	*Acinetobacter junii* *Prevotella oralis* *Prevotella salivae*
N2	71, F	Bitemporal hemianopia	Iso-signal on T1, mixed signal on T2	Null cell	TSS + XRT	Nil	*Corynebacterium pseudodiphthereticum* *Corynebacterium simulans* *Staphylococcus epidermidis* *Staphylococcus lugdunensis*	*Acinetobacter junii* *Prevotella* sp.
N3	54, F	Left homonymous hemianopia	Iso-signal on T1 and T2	LH/FSH	TSS	Nil	*Corynebacterium kroppenstedtii* *Lactobacillus casei* *Lactobacillus rhamnosus*	
N4	41, M	Bitemporal hemianopia	Low signal on T1, Iso-signal on T2 with SSMT	LH,FSH, TSH	TSS	Steroids, thyroxine, growth + sex hormones	*Streptococcus pneumoniae*	
N5	48, F	Asymptomatic	Low signal on T1, mixed signal on T2	Null cell	TSS	Nil	*Staphylococcus epidermidis* *Stenotrophomonas maltophilia*	

*Refers to the predominant cell population found during histopathological assessment

*M* male, *F* female, *SSMT* sphenoid sinus mucosal thickness, *GCS* Glasgow Coma Scale, *TSS* trans-sphenoidal surgery, *XRT* radiotherapy

Two hundred and fifty bacterial clones were subjected to plasmid purification and PCR screening. Overall, differences were observed between the sphenoid sinus microbiota of the PA and NFPA groups (Table 1). Four of the five PA patients harboured atypical respiratory bacterial taxa, including *Enterobacter* sp. and *Escherichia coli*. In contrast, clonal libraries generated from 4 patients with NFPAs comprised predominantly of sequences representative of *Corynebacterium* spp., *Staphylococcus* spp. and *Prevotella* spp. One NFPA patient (Case N4) was found to harbour *Streptococcus pneumoniae* only and had evidence of asymptomatic sphenoid sinus disease preoperatively.

## Discussion

Compositional differences in the sinus microbiota between patients with and without PA have not been reported previously. Our main finding is the presence of *Enterobacter* and *Escherichia* bacterial DNA, from the sphenoid sinuses of PA patients. Bacteria representative of these genera were not identified in non-apoplectic controls.

Investigations of the sinonasal tract have shown members of the corynebacteria, staphylococci and propionibacteria to be well represented in health [5–7]. In the present study, clonal libraries generated from the sinuses of NFPA patients were generally representative of such findings with either *Staphylococcus epidermidis* or *Corynebacterium* spp. predominating following sequencing. In contrast, atypical respiratory pathogens are rarely reported in the context of the heathy upper airways. For example, a recent survey of the nares (n = 1878) reported *E. coli* and *Enterobacter aerogenes* are carried by less than 6.1 and 2.6% of healthy adults, respectively [8]. With regards the sinuses, microbiota studies are generally more limited in terms of sample size, but typically report recovery of these bacterial taxa in less than 5% healthy individuals [7, 9]. Rather, these bacteria are typically associated with infradiaphragmatic sources and have been implicated as potential pathogens in rhinosinusitis following their isolation in symptomatic individuals [10–12]. The clinical significance of *Leuconostoc* carriage by PA patients is unclear and, to the authors knowledge, has not been described in the context of this body site. In the clinical setting, members of this genus are considered recalcitrant to standard microbiological identification, but have been described in the context of opportunistic infection in immunocompromised individuals [13–15].

PA is characterised by a sudden onset of headache and other neurological symptoms and in many patients this is the first sign of a pre-existing pituitary adenoma. Despite the significant patient morbidity associated with this syndrome, the pathophysiology of PA remains poorly understood with a significant proportion of patients presenting with no recognised risk factors [16]. The identification of known respiratory pathogens from the sinuses of PA patients could be indicative of an infective aetiology. In support of this is the observation of increased prevalence of sphenoid sinus mucosal thickening in patients presenting with PA, most notably within the first week of the onset of symptoms [2, 17, 18]. Infection is also a known risk factor for other cerebral vascular phenomena, such as cavernous sinus syndrome [19]. Given the proximity of the sphenoid sinus to the pituitary gland, it is tempting to speculate an infective aetiology for some patients with PA.

On the other hand, it must be noted that in this preliminary study, the patients undergoing pituitary surgery for PA did not exhibit preoperative evidence of sinusitis. Moreover we acknowledge that we did not attempt to culture the bacteria as from previous experience, this can be difficult, in part due to the administration of peri-operative broad-spectrum antibiotics during trans-sphenoidal surgery. Further work is warranted in a larger patient cohort, utilizing next generation sequencing platforms to achieve better microbiota profiling, together with histological analyses of explanted sphenoid sinus and pituitary tissue.

## Conclusion

In this preliminary study, we characterised the sphenoid sinus mucosal microbiota in patients with PA and compared findings to a control group comprising of surgically treated NFPAs. PA patients harboured atypical sinus bacteria, including *Enterobacteriaceae*. In contrast, NFPA patients had a sinus microbiota more representative of health. The abnormal microbiota in PA patients was similar to reported cases of sphenoid sinusitis. Future prospective studies are warranted in order to explore this observation.
